# Shape-sensing robotic-assisted bronchoscopy for pulmonary nodules: initial multicenter experience using the Ion™ Endoluminal System

**DOI:** 10.1186/s12890-021-01693-2

**Published:** 2021-10-16

**Authors:** Michael J. Simoff, Michael A. Pritchett, Janani S. Reisenauer, David E. Ost, Adnan Majid, Colleen Keyes, Roberto F. Casal, Mihir S. Parikh, Javier Diaz-Mendoza, Sebastian Fernandez-Bussy, Erik E. Folch

**Affiliations:** 1 Bronchoscopy and Interventional Pulmonology, Lung Cancer Screening Program, Department of Pulmonary and Critical Care Medicine, Henry Ford Hospital, Wayne State University School of Medicine, 2799 West Grand Blvd, Detroit, MI 48202 USA; 2 Pulmonary Department, Pinehurst Medical Clinic, Pinehurst, NC USA; 3 Pulmonary Department, First Health Moore Regional Hospital, Pinehurst, NC USA; 4 Department of Pulmonary Medicine and Thoracic Surgery, Mayo Clinic, Rochester, MN USA; 5 Department of Pulmonary Medicine, The University of Texas MD Anderson Cancer Center, Houston, TX USA; 6 Department of Thoracic Surgery and Interventional Pulmonology, Beth Israel Deaconess Medical Center, Harvard Medical School, Boston, MA USA; 7 Department of Pulmonary and Critical Care Medicine, Massachusetts General Hospital, Harvard Medical School, Boston, MA USA; 8 Department of Pulmonary Medicine, Mayo Clinic, Jacksonville, FL USA

**Keywords:** Pulmonary nodules, Biopsy, Shape sensing, Bronchoscopy, Robotic assistance, Ion

## Abstract

**Background:**

Traditional bronchoscopy provides limited approach to peripheral nodules. Shape-sensing robotic-assisted bronchoscopy (SSRAB, Ion™ Endoluminal System) is a new tool for minimally invasive peripheral nodule biopsy. We sought to answer the research question: Does SSRAB facilitate sampling of pulmonary nodules during bronchoscopists’ initial experience?

**Methods:**

The lead-in stage of a multicenter, single-arm, prospective evaluation of the Ion Endoluminal System (PRECIsE) is described. Enrolled subjects ≥ 18 years old had recent computed tomography evidence of one or more solid or semi-solid pulmonary nodules ≥ 1.0 to ≤ 3.5 cm in greatest dimension and in any part of the lung. Subjects were followed at 10- and 30-days post-procedure. This stage provided investigators and staff their first human experience with the SSRAB system; safety and procedure outcomes were analyzed descriptively. Neither diagnostic yield nor sensitivity for malignancy were assessed in this stage. Categorical variables are summarized by percentage; continuous variables are summarized by median/interquartile range (IQR).

**Results:**

Sixty subjects were enrolled across 6 hospitals; 67 nodules were targeted for biopsy. Median axial, coronal and sagittal diameters were < 18 mm with a largest cardinal diameter of 20.0 mm. Most nodules were extraluminal and distance from the outer edge of the nodule to the pleura or nearest fissure was 4.0 mm (IQR: 0.0, 15.0). Median bronchial generation count to the target location was 7.0 (IQR: 6.0, 8.0). Procedure duration (catheter-in to catheter-out) was 66.5 min (IQR: 50.0, 85.5). Distance from the catheter tip to the closest edge of the virtual nodule was 7.0 mm (IQR: 2.0, 12.0). Biopsy completion was 97.0%. No pneumothorax or airway bleeding of any grade was reported.

**Conclusions:**

Bronchoscopists leveraged the Ion SSRAB’s functionality to drive the catheter safely in close proximity of the virtual target and to obtain biopsies. This initial, multicenter experience is encouraging, suggesting that SSRAB may play a role in the management of pulmonary nodules.

*Clinical Trial Registration identifier and date* NCT03893539; 28/03/2019.

## Background

Chest imaging has seen significant growth in the United States and worldwide due to its non-invasive ability to detect pulmonary conditions. In the United States more than an estimated 1.6 million nodules are detected each year as incidental findings on chest radiographs and computed tomography (CT) scans [1]. Also seen is increased identification of nodules due to the growth in lung cancer screening programs based on low-dose CT scans to assess patients who are high risk for lung cancer. Such screening has resulted in an 8–51% incidence of solitary pulmonary nodules within selected populations [2]. While most nodules may require surveillance, a significant number require tissue biopsy.

Because of their larger diameters, standard bronchoscopes cannot progress beyond the subsegmental bronchi and consequently provide a limited approach to peripheral nodules [3]. The overall sensitivity for malignancy of flexible bronchoscopy is 34% for lesions < 2 cm and 63% for > 2 cm [4]. The development of virtual bronchoscopy, fluoroscopic guidance, radial endobronchial ultrasound (rEBUS), ultrathin bronchoscopes, and electromagnetic navigation bronchoscopy (ENB) for the diagnosis of peripheral nodules has improved our ability to sample smaller and more peripheral lesions. Yet, a meta-analysis resulted in a pooled diagnostic yield (based on these techniques prior to 2010) evidenced 60.9% for lesions ≤ 2 cm—demonstrating the continued challenge to approaching small peripheral nodules [5, 6]. Two recent studies—a real-world single-arm cohort and a meta-analysis, respectively—described a 73% yield from ENB for nodules with a median size of 2 cm and a pooled sensitivity for malignancy of 77% with a good safety profile with an average lesion size of 23.2 mm [7, 8]. Such reports highlight the need for improved procedural outcomes for small peripheral nodules by developing new bronchoscopy tools and approaches, while maintaining a low complication rate.

A shape-sensing robotic-assisted bronchoscope (SSRAB) is a new tool for minimally invasive peripheral nodule biopsy. The Ion™ Endoluminal System (Intuitive Surgical, Inc., Sunnyvale, CA) received its 510 K from the FDA in 2019. In the first human use study of a pre-commercial iteration of the Ion Endoluminal System, targets with a mean size of approximately 14 mm were reached in 96.6% of cases; the overall diagnostic yield was 79.3% with no reported incidence of pneumothorax or bleeding [9].

Does SSRAB facilitate sampling of pulmonary nodules in human subjects during bronchoscopists’ early experience? The authors hypothesize that SSRAB does facilitate biopsy of pulmonary nodules, including small and peripherally based nodules, in these subjects. This manuscript is a report of the initial multicenter experience in human subjects, including procedural characteristics and descriptive outcomes. [ClinicalTrials.gov identifier and date: NCT03893539; 28/03/2019].

## Methods

The current study describes the initial or lead-in stage (Stage 0) of the larger PRECIsE study—a multistage, single-arm, prospective evaluation of the SSRAB System to bronchoscopically approach and facilitate sampling of pulmonary nodules. The purpose of this lead-in stage was to provide participating investigators and support staff their first human experience with the SSRAB system and its associated workflow. The study period (date of first enrollment to last follow up) was from 29 March, 2019 to 10 January, 2020. For the lead-in cases, subjects were followed to and not beyond 30 days post procedure. The focus was collection of early complications and safety data. The performance metrics of yield and sensitivity will be addressed and analyzed in subsequent publications.

Pre-specified enrollment in this initial stage was limited to 10 subjects per each of the six participating centers, with at least 5 subjects per bronchoscopist. Enrolled subjects were ≥ 18 years old, were suitable candidates for elective bronchoscopy, had recent evidence on CT of one or more solid or semi-solid pulmonary nodules ≥ 1.0 to ≤ 3.5 cm in greatest dimension and in any part of the lung. Subjects considered for enrollment in this study had a moderate-to-high risk of malignancy; high-risk subjects were enrolled if they wanted diagnostic confirmation prior to treatment. Subjects with a suspicion of metastatic disease were also considered; all considered subjects included those for whom investigators would consider further interventions if indicated to confirm diagnosis. Each center obtained institutional review board (IRB) approval (study sites, IRB committee names, and approval numbers follow. Mayo Clinic: Mayo Clinic IRB 18-011348; First Health Moore Regional Hospital: Western IRB 20183121; Massachusetts General Hospital: Dana Farber Cancer Institute IRB 19-209; Beth Israel Deaconess Medical Center: Dana Farber Cancer Institute IRB 19-209; Henry Ford Hospital: Henry Ford Health System IRB 12822; University of Texas MD Anderson Cancer Center: MD Anderson Cancer Center IRB IRB00006023). Enrolled study participants provided written informed consent, and study subject confidentiality was maintained according to the Health Insurance Portability and Accountability Act requirements.

**Fig. 1:**
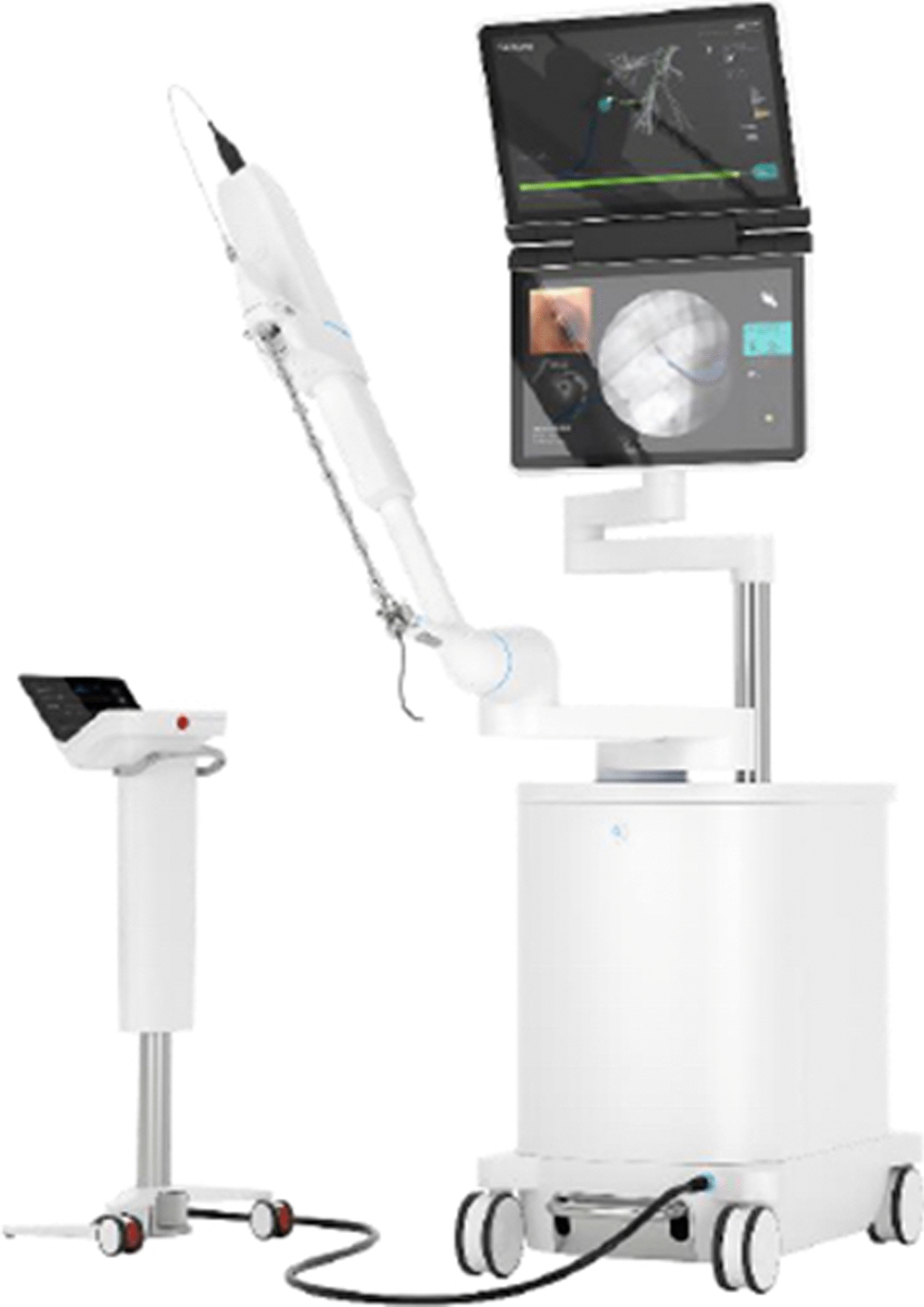
Ion Endoluminal System. All rights reserved; used with permission from Intuitive Surgical

**Fig. 2:**
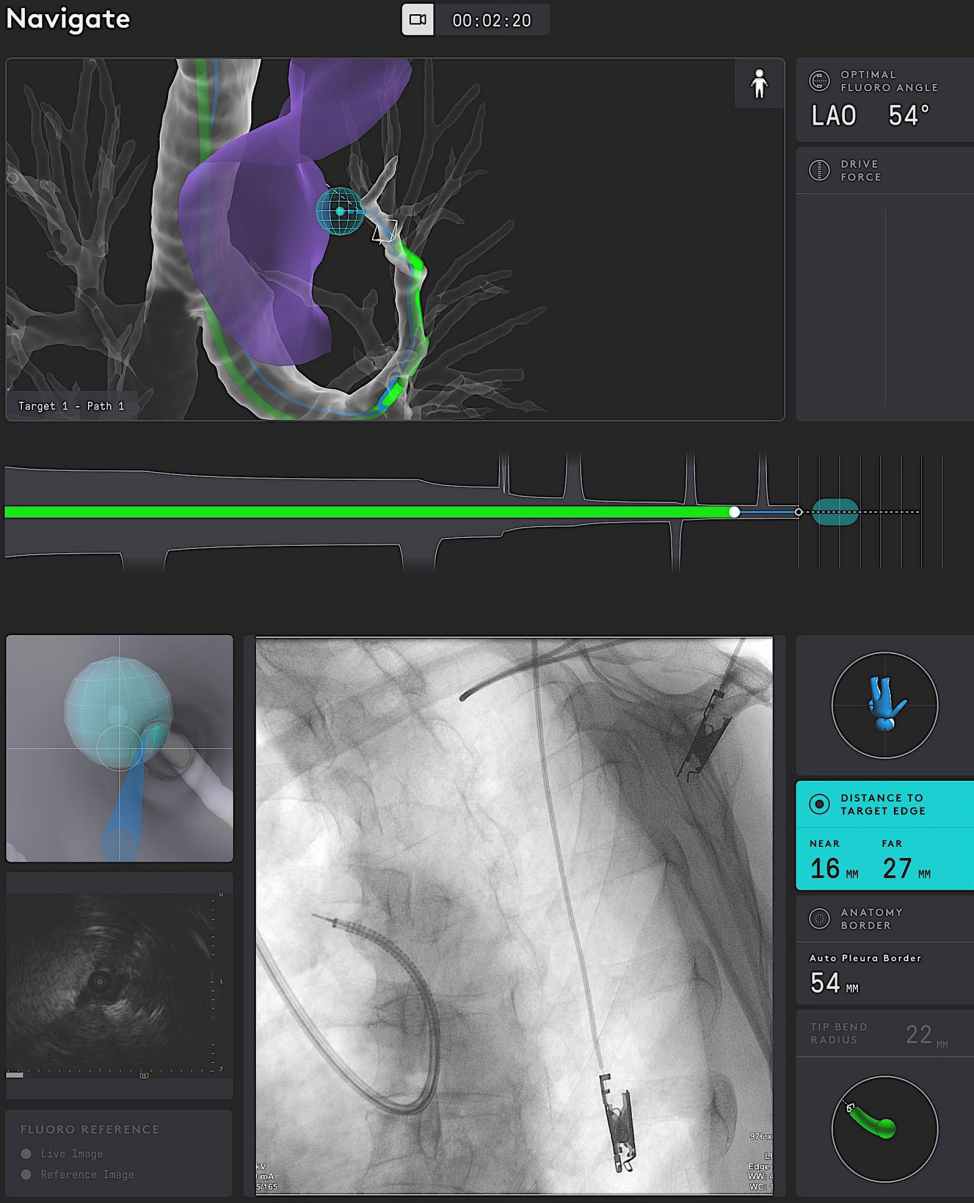
Procedural screenshot from one case. *Top monitor screens:* virtual airway view showing catheter within airway tree; blue ball represents target; green subway view at bottom shows the progress of the catheter to the target; drive force displayed on the right. *Bottom monitor screens:* (upper left) virtual target with target view; (bottom left) integrated EBUS view; (center) fluoroscopy view of catheter with tool extension; (right side) informational screen displaying distance to virtual target, anatomy borders, orientation guide, and catheter tip bend radius

**Fig. 3:**
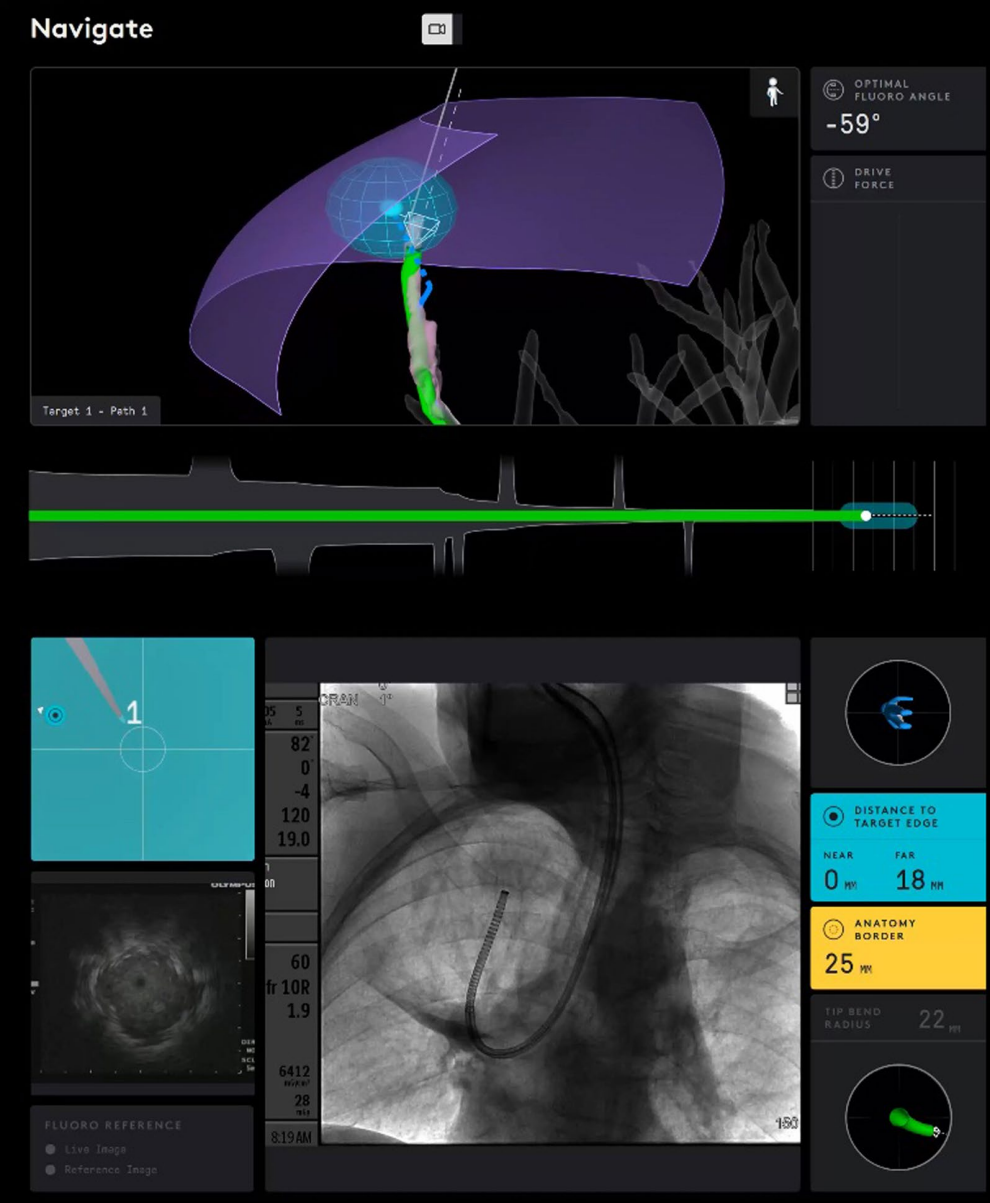
Another screenshot from a case showing (top) the catheter reaching the virtual target and (bottom) fluoroscopic view of catheter bend to the apical lesion

**Fig. 4:**
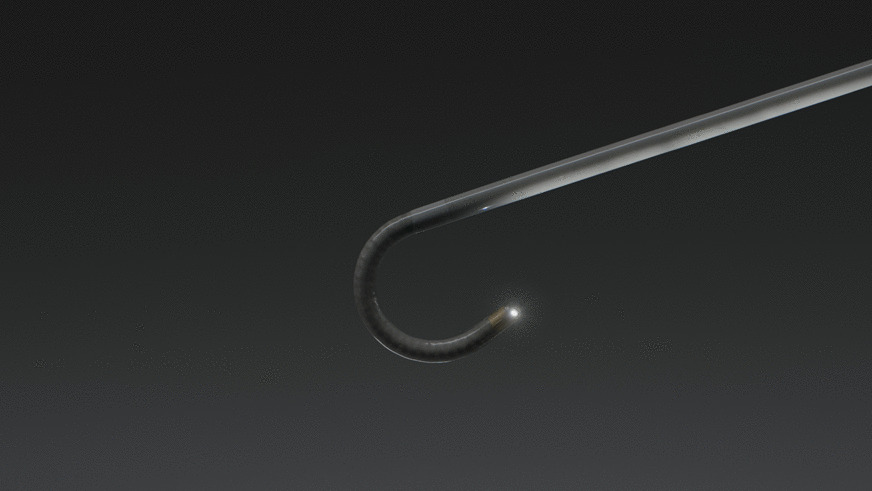
Catheter instrument (3.5 mm outer diameter) with vision probe in articulated position. All rights reserved; used with permission from Intuitive Surgical

**Fig. 5:**
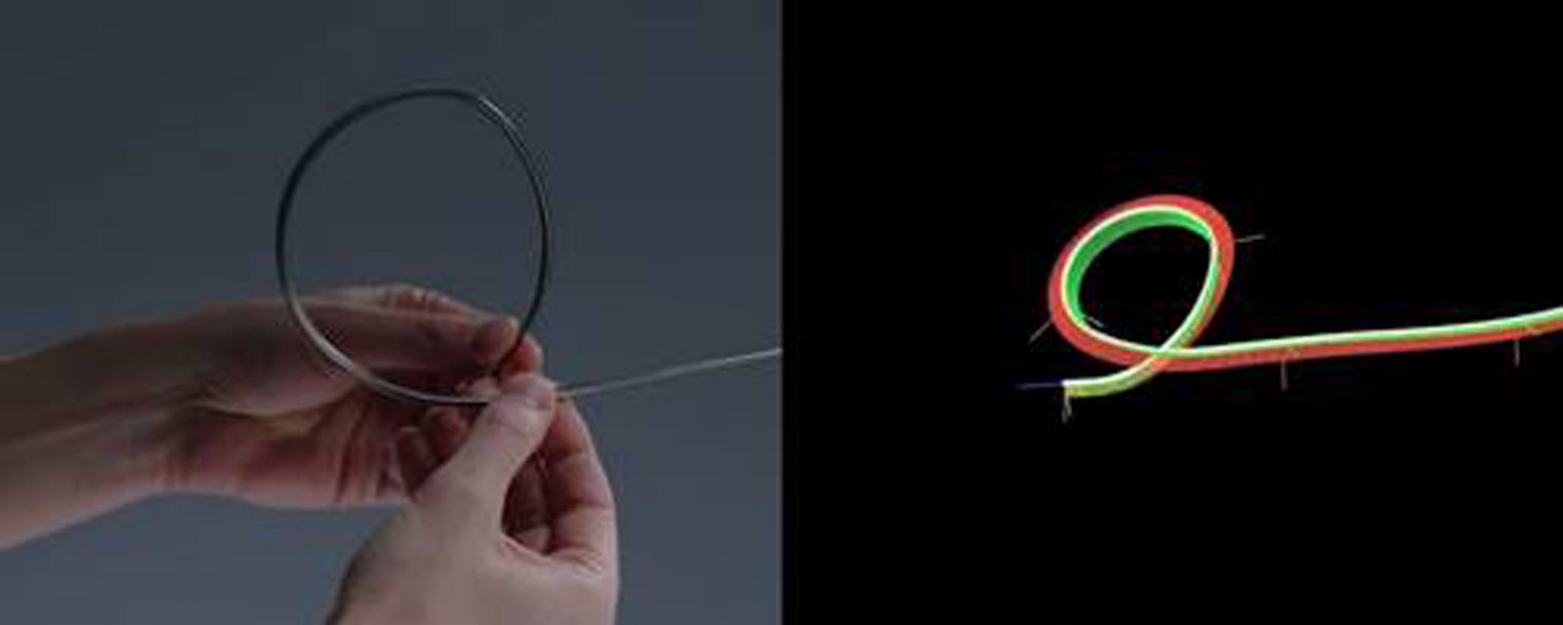
Image illustrating shape-sensing technology along catheter’s entire length. All rights reserved; used with permission from Intuitive Surgical

Briefly described, the SSRAB system is comprised of a robotic system cart, controller and fully articulating catheter instrument with embedded shape-sensing capabilities (Fig. 1). The robotic system cart facilitates the movement of the catheter instrument via the instrument arm by translating input from the standalone controller and leverages a pull-wire system to drive the catheter into the airways under direct visualization provided by a vision probe. The cart houses two system monitors that provide visual information including: virtual and live airway views, the airway tree with catheter position overlaid, target information, and third-party video sources such as radial endobronchial ultrasound (rEBUS)and fluoroscopy (Figs. 2 and 3). The fully articulating catheter is 3.5 mm in outer diameter with a 2-mm working channel and a steerable distal tip, which can be articulated up to 180 degrees in any direction (Fig. 4). A thin flexible fiber, which provides the basis for the shape-sensing technology, is embedded along the catheter’s entire length and measures its own shape hundreds of times per second, representing and displaying throughout the procedure the shape and position of the catheter relative to the anatomy (Fig. 5). The catheter also provides feedback to the robotic control algorithm that maintains the intended position for the catheter instrument, enabling a fixed and stable position and correcting for any deflections from the intended position by providing extra force through the appropriate pull-wire system. The technology and instruments are immune to electromagnetic interference and are unaffected by metallic objects or electromagnetic fields.

Each investigator completed standardized training, which allowed bronchoscopists to gain experience and basic familiarity with the system through in-depth dry lab in-service training, a porcine lab, and a cadaveric lab. In-service training was completed prior to any clinical cases primarily for support staff and included assistance with room setup and process workflow.

**Fig. 6:**
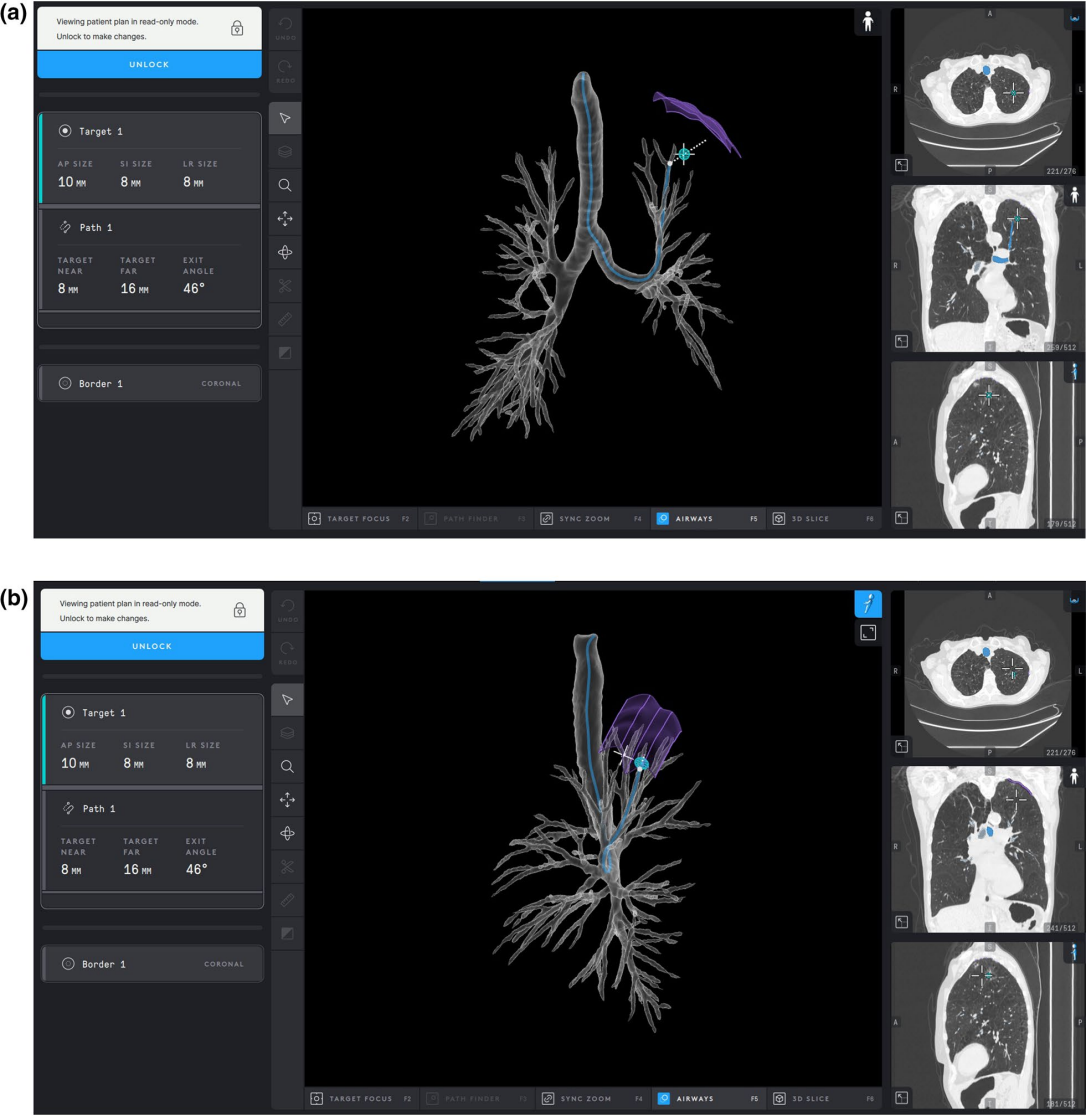
**a, b** Segmented CT scans of the same subject from PlanPoint planning software from two orientations, both of which show the target nodule, identified pathway, and anatomy border

Pre-procedure CT scans (0.75–1.25 mm slice thickness) were segmented using the system-specific PlanPoint™ planning software with pathway planning completed and reviewed through a virtual simulation (Fig. 6a and Fig. 6b). With the patient under general anesthesia, standard airway examinations were initially performed using a flexible bronchoscope and included clearing of secretions and review of the normal anatomy to be navigated. The SSRAB system was then docked to the endotracheal tube via a magnetic adapter. After registration, navigation of the catheter to the target nodule was completed under direct visualization and in accordance with the virtual plan created using the pre-procedure CT. When the target was reached, the vision probe was removed and a combination of rEBUS and fluoroscopy were used to assess real-time information regarding the nodule and to refine the biopsy location. After appropriate adjustments were made to the catheter position, sampling commenced using the system-specific flexible needle (Flexision™, 19G, 21G, or 23G) using the cloud biopsy technique. Cloud biopsy, which describes the systematic and consistent placement and/or redirection of biopsy tools in specific sampling areas, was enabled by the fine manipulation of the scope tip in any plane to optimize the angle towards the target for further biopsies. The cloud biopsy facilitates the collection of samples from four different quadrants of a target nodule. The bronchoscopist was able to perform this technique due to the ability to make micro adjustments of the catheter tip to optimize the biopsy zone. Forceps, cytology brush, and/or bronchioalveolar lavage were used at the bronchoscopist’s discretion. Rapid on-site cytology evaluation (ROSE) was available at 5 of 6 participating centers and was used according to institution practice and was not standardized. Following the procedure, a fluoroscopic check for pneumothorax was performed; in the same anesthesia event, EBUS-TBNA staging (where indicated) was performed. A chest x-ray was taken at least one-hour post-procedure to assess for delayed pneumothorax.

Both the nodule size (assessed in axial, coronal and sagittal planes) as well as the distance of the closest edge of the target nodule to the closest pleura or fissure were both based on pre-procedure CT scans. Bronchial generation count was assessed with the trachea representing generation 0 and each subsequent carina or bifurcation/trifurcation counting as an additional generation based on the pre-procedure CT and typically using the segmented model. Each bronchoscopist assessed the nodule characteristics including presence of bronchus sign on pre-procedure CT and rEBUS visualization during the procedure according to individual practice. Registration time was the total time necessary to complete registration prior to the start of navigation; biopsy time (using SSRAB for the peripheral nodule) was the time from first biopsy tool insertion to last peripheral biopsy tool withdrawn. Procedure time specifically for the SSRAB system was measured from time of catheter insertion to withdrawal; durations of airway survey prior to the use of Ion nor the duration of further diagnostic procedures, including EBUS-TBNA, were not included in these times. Use of a PlanPoint-generated path reflected whether the bronchoscopist followed a system-generated pathway to the virtual target nodule. Catheter positions represented the unique parked locations of the catheter tip in relation to the nodule. Tool passes reflected the total number of biopsy tools passed through the catheter, with each tool counted separately and no reliance on sample acquisition. Distance between the catheter tip and the closest edge of the virtual target when the catheter was parked prior to biopsy was displayed on the system cart monitor. Adverse events related to the bronchoscopy procedure were collected, including any type of pneumothorax and bleeding that did not stop spontaneously or that required intervention. Subjects were followed at 10 days and 30 days post-procedure.

The purpose of this stage of the study was to provide participating investigators and support staff their first human experience with the SSRAB system; safety and procedure outcomes were analyzed. Diagnostic yield or sensitivity for malignancy were not assessed in this stage. Data in the current analysis will not be used in subsequent multicenter outcomes analyses. Subject and procedure outcomes for this lead-in stage are presented descriptively; categorical variables are summarized by percentage; continuous variables are summarized by median/IQR. The statistical software package SAS® version 9.4 (SAS Institute, Cary, NC, USA) was used.

## Results

Sixty subjects were enrolled across the six participating institutions, and a total of 67 nodules were targeted for biopsy. The study protocol recommended the timing between the pre-procedure CT scan and the procedure day not to exceed 21 days. On average, the CTs were taken approximately 5 days prior to the procedures and approximately 30% of the enrolled subjects had their CT scans the day of their procedures. Demographics are described in Table 1. The median pretest probability of malignancy using the Mayo/Swensen model was 46.3% (IQR: 23.8, 77.3) [9]; the majority (76.7%) of subjects were classified as American Society of Anesthesiologists (ASA) III.

**Table 1:** Baseline characteristics of subjects (n = 60 subjects)

Variable	Outcome
Age, median, y (IQR)	70.7 (63.1, 76.6)
BMI, median kg/m^2^ (IQR)	25.0 (22.9, 31.6)
Gender, n (%)	
Female	35 (58.3)
Male	25 (41.7)
Pretest probability of malignancy, median % (IQR) ^a^	46.3 (23.8, 77.3)
ASA class, n (%)	
II	14 (23.3)
III	46 (76.7)

*IQR* interquartile range, *BMI* body mass index, *ASA* American Society of Anesthesiologists

^a^Swensen’s Formula: low risk < 5%; indeterminate risk 5–65%; high risk > 65%; from reference [9]

Nodule characteristics are provided in Table 2. Median axial, coronal, and sagittal dimensions were each 17.5, 16.0, and 16.2 mm, respectively, and the median largest cardinal diameter was 20.0 mm. Approximately half (52.3%) of nodules were located in the upper lobes and 82.1% of nodules were solid. Nodules were predominately extraluminal as assessed visually and the median distance from the outer edge of the nodule to the nearest pleura or fissure was 4.0 mm (IQR: 0.0, 15.0), with approximately 31% in contact with the pleura or fissure as measured on the pre-procedure CT scan. A bronchus sign was present for 32.7% of nodules. Median bronchial generation count was 7.0 (IQR: 6.0, 8.0).

**Table 2:** Nodule characteristics (n = 67 nodules)

Variable	Outcome
Size, median mm (IQR)	
Axial	17.5 (12.0, 24.0)
Coronal	16.0 (11.8, 21.0)
Sagittal	16.2 (12.0, 22.0)
Largest cardinal diameter	20.0 (14.0, 27.0)
Lobe location, n (%)	
LLL	8 (11.9)
LUL	18 (26.9)
RLL	21 (31.3)
RML	3 (4.5)
RUL	17 (25.4)
Distance from pleura or fissure, median mm (IQR)	4.0 (0.0, 15.0)
Bronchial generation count, median n (IQR)	7.0 (6.0, 8.0)
Location, n (%)^a^	
Endoluminal	10 (15.4)
Extraluminal	55 (84.6)
Nodule type, n (%)	
Solid	55 (82.1)
Cavitary	4
Semi-solid	12 (17.9)
CT Bronchus sign present, n (%)	25 (37.3)
rEBUS attempted, n (%)	66 (98.5)
rEBUS visualization, n (%)^b^	59 (89.4)
Concentric, initially	23 (40)
Eccentric converted to concentric	8 (13.6)
Eccentric	28 (47.5)

*IQR* interquartile range, *LLL* left lower lobe, *LUL* left upper lobe, *RLL* right lower lobe, *RML* right middle lobe, *RUL* right upper lobe, *rEBUS* radial endobronchial ultrasound

^a^2 subjects had early termination

^b^rEBUS visualization is based on nodules attempted

Procedural characteristics, including the median duration of each stage of the procedure, are provided (Table 3). Navigation planning pre-procedure was 10.0 min (IQR: 5.0, 15.0), and the median duration of the procedures from catheter in–to catheter out was 66.5 min (IQR: 50.0, 85.5) including sampling of multiple nodules within the same subject (7 cases, each with 2 nodules biopsied) as well time associated with ROSE results and/or obtaining multiple samples. ROSE was completed in 78.3% of cases. Once registration was completed, navigation to the first nodule took 5.0 min (IQR: 3.0, 10.0). In nearly all (93.1%) of the cases, the PlanPoint path was used to reach the target nodule. During the biopsy sequence using a needle first, forceps, and/or cytology brush, there was an average of four catheter tip positions. There was a median of 13 biopsy tool passes per procedure, and rEBUS was used for 66 out of 67 nodules. rEBUS was not attempted in two cases due to technical due to technical or clinical reasons at the discretion of the proceduralist. The remaining five nodules were in the lower lobe, and three did not have CT bronchus sign. The median time to achieve first rEBUS visualization was 8 min from navigation start. The use of rEBUS achieved visualization of 89.4% of nodules where rEBUS was attempted; of nodules visualized with rEBUS, an initial concentric view was obtained in 39% of visualized nodules, while an additional 13.6% of nodules that were initially found to have an eccentric view became concentric after further adjustment and creation of a path to the nodule.

**Table 3:** Procedural characteristics (n = 60 subjects)

Variable	Result
Duration, median min (IQR)	
Navigation planning	10.0 (5.0, 15.0)
Procedure (scope in to scope out)	66.5 (50.0, 85.5)
Registration	8.5 (5.0, 14.0)
Navigation to 1st nodule	5.0 (3.0, 10.0)
Biopsy completion	31.5 (25, 46.5)
Lymph node staging performed, n (%)	47 (78.3)
PlanPoint path used, n (%)	54 (93.1)
Time to 1st rEBUS visualization, min (IQR)	8.00 (4.0, 13.5)
Catheter positions, n (IQR)	4.0 (2.0, 7.0)
Tool passes, median n (IQR)	13.0 (8.0, 15.0)
Tools used, n (%)^a,b^	
Needle^c^	58 (100)
Forceps	40 (69)
Brush	28 (48.3)

*IQR* interquartile range

^a^Tools used out of 58 completed procedures; 2 subjects with early termination not included

^b^More than one tool may have been used per case

^c^System-specific Flexision needle

Biopsy completion, whereby a tool was passed through the catheter and a tissue sample obtained, was 96.7% for all subjects and 97.0% for all nodules (Table 4). Among completed biopsies, the median distance from the catheter tip to the edge of the virtual target was 7.0 mm (IQR: 2.0, 12.0). There were two subject cases where biopsies were not attempted and an alternative method was used. In one case, the CT slice thickness was incompatible for registration; the case was completed using bronchoscopy with rEBUS. In the other case, the physician was unable to reach the nodule due to the lack of a patent airway and the procedure was completed using an esophageal approach using a cEBUS scope (EUS-B).

**Table 4:** Biopsy outcomes

Variable	Outcome
Biopsy completion, n (%)^a^	
Subject level (n = 60)	58 (96.7) 95% CI: 92.1%, 100%^b^
Nodule level (n = 67)	65 (97.0) 95% CI: 92.9%, 100%^b^
Closest distance to nodule, median mm (IQR)^c^	7.0 (2.0, 12.0)
Serious adverse events, n (%)	0 (0)
Pneumothorax (with or without chest tube)	0 (0)
Airway bleeding (with or without intervention)	0 (0)
Other	0 (0)
Complications, n (%)	2 (3.3)
Cardiac arrhythmia, CTCAE grade 2 (intra-procedure)	1 (1.7)
Pneumonia, CTCAE grade 2 (post-procedure)	1 (1.7)

*IQR* interquartile range, *CI* confidence interval

*CTCAE* Common Terminology Criteria for Adverse Events; from reference 11

^a^Biopsy completion = tool passed through catheter and a sample was obtained

^b^95% Confidence Interval calculated using the Wald method

^c^Closest distance from tip of catheter to edge of the virtual nodule

No serious adverse events were reported, including no pneumothorax or airway bleeding of any grade [10, 11]. Two complications were reported, both of which were classified as Common Terminology Criteria for Adverse Event (CTCAE) grade 2 [12]. One subject experienced cardiac arrhythmia during the procedure, which was treated with medication with anesthesia and resolved immediately. The subject did not have any symptoms post-procedure and was discharged. Another subject developed pneumonia 48 h post-procedure and was treated with oral antibiotics at home with resolution of symptoms after approximately 10 days. Neither complication was attributed to the SSRAB system.

## Discussion

Although the standard of care for the majority of incidentally found pulmonary nodules is active surveillance, any nodule greater than 8 mm with a moderate pretest probability potentially warrants biopsy [13]. The introduction of new technologies, such as ENB and rEBUS have advanced bronchoscopy, but despite these technologies and tools, the reported diagnostic yield for peripheral nodules < 2 cm is 40–67% [1, 7, 14, 15].

The early data from pre-clinical studies and the single-center first human use experience with SSRAB suggest that this very stable and sensitive platform allows bronchoscopists to guide tools and localize peripheral tumors to a higher degree than was previously possible [2]. The lead-in results for the larger PRECIsE Study of the Ion Endoluminal System are the earliest cases performed at six centers by eleven physicians, including both interventional pulmonologists and thoracic surgeons. None of the physicians had used the SSRAB system prior to these cases, and this initial series was limited to the first 10 cases for each center or the first 5 cases each for centers with more than one bronchoscopist.

**Fig. 7:**
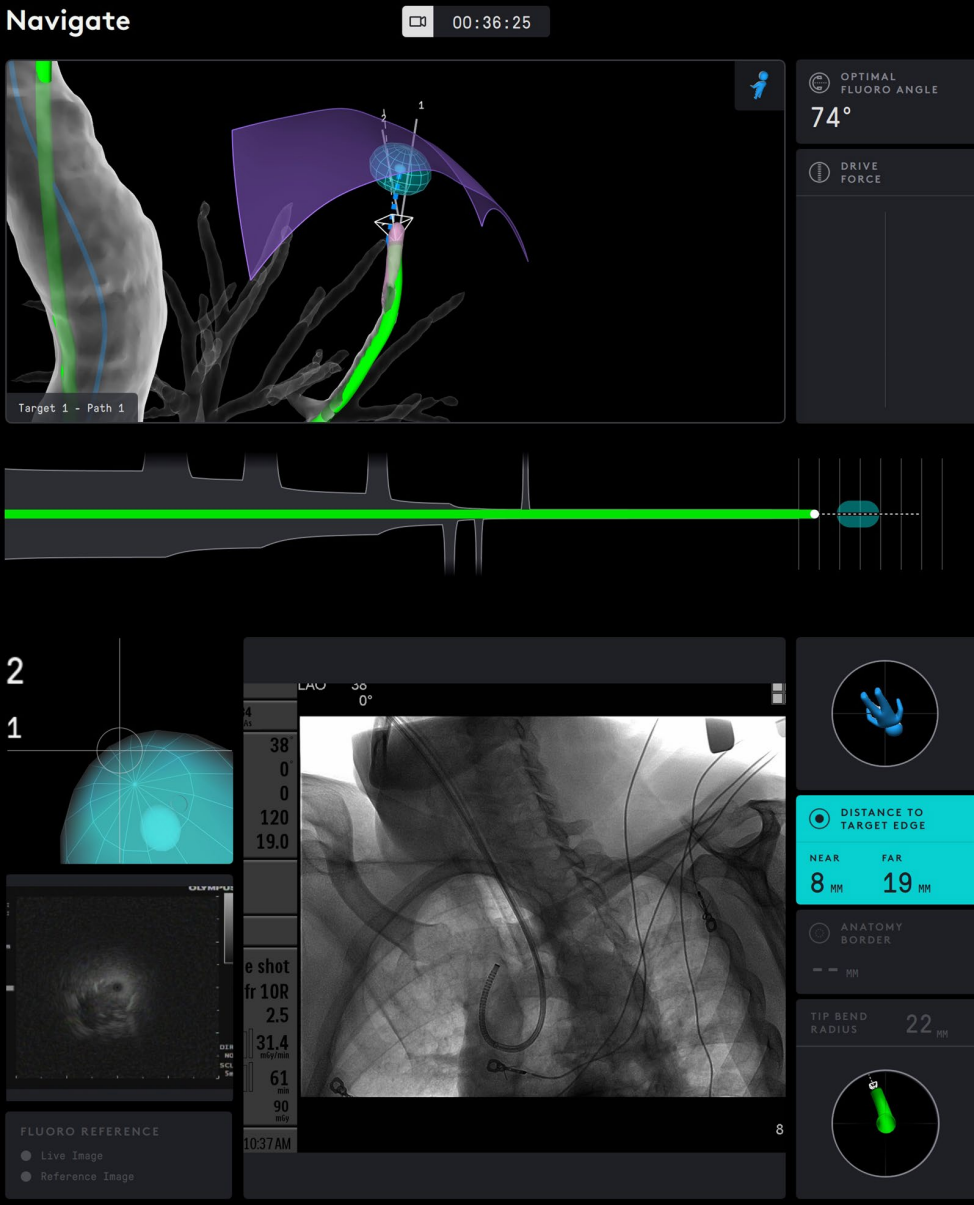
Display of catheter on the system monitors during biopsy. The catheter position is shown in green and is interlaid on the virtual airway map. On the bottom screen, fluoroscopy reveals the tight bend of the catheter through the airways to reach the target nodule. Distance to the nearest and farthest edge of the target nodule is displayed at the lower right corner

Small peripheral nodules, which historically have low diagnostic yields, were targeted. The SSRAB technology facilitated navigation to this type of nodule. Despite the introductory nature of the technology for the bronchoscopists, they approached nodules with a median axial size < 18 mm. The ability to drive the catheter to the target and then fix the catheter in location allowed passage of rEBUS, a system-specific flexible biopsy needle, forceps, or brushes without deviation from the target. Significant in each case was the ability of the Flexision needle to traverse very tight angles into peripheral airways allowing for repeat sampling at the same location, as illustrated in Fig. 7. This initial experience suggests the investigators were able to target small peripheral lesions during the first few cases, expeditiously and safely; however, diagnostic sensitivity and diagnostic yield metrics associated with nodules of this size warrants further investigation. While the clinical utility of the system will be reported in future publications, in most cases, EBUS lymph node staging was performed after biopsy during the same procedure in order to potentially reduce CT-body divergence (single anesthetic event) optimizing workflow for comprehensive diagnosis of suspicious nodules with complete staging in a single procedure [16].

In an operating room or bronchoscopy suite, each minute is a unit of time that influences personnel use and direct costs. Our cases had a median procedure time of 66.5 min and total biopsy workflow time of 31.5 min. Folch et al. recently reported data from a large multicenter evaluation of ENB, 92.2% of whom performed > 5 ENB procedures per month with experienced ENB teams [7]. Their median total procedure time was 52 min and median total ENB-specific procedure time was 25 min. Given the established teams and navigational platforms in their reported study, we are encouraged by our initial experience and anticipate comparable procedural times using the SSRAB system in the subsequent phase of the PRECIsE study.

Because the unique shape-sensing feedback (rather than electromagnetic navigation) is fundamental to the Ion system, biopsy tools can be introduced through the catheter and simultaneous use of fluoroscopy can be incorporated throughout the procedure without affecting navigational accuracy. The real-time feedback allows for frequent referencing to the virtual target throughout the procedure and does not require movement of equipment into and/or out of the procedure field, providing for continuity in procedural workflow and facilitating stability of the catheter when positioned for biopsy. The physician can potentially correct for perceived CT-to-body divergence during navigation by identifying airways on the camera image and subsequently comparing them to the virtual image. If the physician believes the catheter is in the wrong airway, alternative airways can be quickly identified and accessed. Similarly, the virtual nodule position can be adjusted for rEBUS and fluoroscopic input, allowing an organized, accurate biopsy procedure.

With one-third of the nodules at least 8 generations out, the investigators perceived that SSRAB accessed nodules in peripheral locations. With the nodule identified, the combination of catheter size and flexibility, real-time shape feedback, and live visualization during navigation facilitated not only reaching within 7.0 mm of the virtual target but also passage of biopsy tools, including the Flexision biopsy needle, through any bend of the catheter to obtain tissue samples from the intended location. These outcomes suggest SSRAB will contribute significantly to the management of suspicious peripheral nodules.

Sampling, using the flexible needle, forceps, and/or brush biopsy tools, was performed with virtual targeting, fluoroscopy, and rEBUS. The shape-sensing technology detected and corrected for any catheter tip deflection that occurred from the insertion or removal of the rEBUS catheter or biopsy tools. By leveraging the capability to perform cloud biopsies, the broncoscopist can use the virtual, fluoroscopic, and ultrasound images to further localize the lesion for additional sampling, updating the position of the virtual target based on the gained real-time information. That the nodule visualization rate using rEBUS was 89.4% at the terminus of navigation demonstrates the initial localization success that SSRAB may provide, despite the majority of nodules not having a bronchus sign on pre-procedure CT. Early rEBUS literature reported visible lesions in approximately 67% of cases while more recent studies have reported an increase to 96% [17, 18]. rEBUS visualization characteristics and determination of true nodule identification versus artifact or atelectasis interpreted as nodule visualization is being studied [19]. Future studies may benefit from a standardized definition and review of imaging to confirm rEBUS visualization and characteristics.

Despite the high biopsy rate (96.7%) in the current study, there were no events of pneumothorax or hemorrhage. This compares with the full cohort in the NAVIGATE study where the overall pneumothorax rate was 4.3% and, for those pneumothorax graded CTCAE ≥ 2, the rate was 2.9%. The ENB-related bronchopulmonary hemorrhage rate graded CTCAE ≥ 2 was 1.5% [7]. The encouraging safety profile should be noted in the context of this series describing the first human use of this technology for each bronchoscopist and suggests this technology’s comparable safety profile to guided bronchoscopic approaches and significantly improved over percutaneous biopsy approaches. While encouraging, safety speculation is based on a limited number of subjects and further safety data from a larger cohort is forthcoming. As a remote-manipulator system, one of the limitations of robotic systems is the lack of haptic feedback that the physician may be accustomed to when performing manual bronchoscopy. During our initial experience, the use of visual cues provided feedback and confidence during navigation and sampling, supported by the low complication rate and the absence of observed airway trauma. Future publications will evaluate the sensitivity of malignancy and yield associated with this system relative to other approaches to further contextualize the value of Ion’s safety profile.

Given the lead-in nature of this study, it has inherent limitations. All of the bronchoscopists and their teams were new to SSRAB technology in live cases and, thus, the described data were gained early in their learning curve. However, because they are highly experienced with bronchoscopic procedures, including with other navigation platforms, their experiences with SSRAB may not reflect the real world of bronchoscopists who may be early in their interventional pulmonary practice. As with any new technology, the use of SSRAB, its integration into the workflow, and the experience of the bronchoscopist and team all can affect durations and outcomes. Despite the fact that each center and physician had significant experience with ENB and other advanced diagnostic approaches, a standardized training program with mentorship provided by technical staff was completed prior to the first human experience. Performance metrics such as yield and sensitivity were not assessed due to limited follow-up, however performance metrics met the purpose of the lead-in phase. Further study is needed to evaluate performance. Other limitations include the enrollment of subjects according to controlled eligibility criteria, although the study’s eligibility criteria modeled the population typically indicated for this type of biopsy procedure. Furthermore, the specific biopsy sequence and tool usage, as well as assessment of characteristics of CT bronchus sign or rEBUS visualization was not standardized: the view—eccentric or concentric—was determined by physician assessment. This was by design a goal of this lead-in stage to understand the real-world workflow and experience associated with this new technology. Last, the intent of the current single-arm analysis was generation of evidence regarding early experience. Future comparative studies should be considered in those centers where experience and proficiency with current technologies have been obtained. Strengths of the study are its multicenter design and the prospective collection and reporting of data from each bronchoscopist’s first cases.

## Conclusions

In this early experience with the Ion Endoluminal System and its shape-sensing navigation technology, bronchoscopists were successful in their ability to safely drive the catheter tip within close proximity of the virtual target for peripheral nodules, and they were able to obtain one or more biopsy samples of small, peripherally based nodules and—when necessary—perform lymph node staging within the same procedure. Both site and research-specific experiences have led to modified approaches and workflows with SSRAB, setting the stage for the full prospective evaluation, which will be reported at study completion. This initial, multicenter experience is encouraging and suggests the significant role SSRAB may play in the management of pulmonary nodules.

## Data Availability

The datasets generated and/or analyzed during the current study are not publicly available because the current study describes the initial analysis, which is not inclusive of the overall results of the PRECIsE Study; the datasets are available from the corresponding author upon reasonable request.

## Abbreviations

CT: Computed tomography
CTCAE: Common terminology criteria for adverse event
ENB: Electromagnetic navigation bronchoscopy
EUS-B: Transesophageal ultrasound/endobronchial ultrasound
IQR: Interquartile range
IRB: Institutional review board
rEBUS: Radial endobronchial ultrasound
ROSE: Rapid on-site cytology evaluation
SSRAB: Shape-sensing robotic-assisted bronchoscopy
TBNA: Transbronchial needle aspiration
